# Three-Random-Point Marking Method for Toric Intraocular Lens Alignment Using the iTrace Aberrometer

**DOI:** 10.1155/2021/6695454

**Published:** 2021-04-08

**Authors:** Yongyi Niu, Hongliang Lin, Yongjie Qin, Cheng Yang, Yanlei Chen, Jin Zeng, Hongyang Zhang

**Affiliations:** ^1^Department of Ophthalmology, Guangdong Eye Institute, Guangdong Academy of Medical Sciences, Guangdong Provincial People's Hospital, Guangzhou, China; ^2^Southern Medical University, Guangzhou, China; ^3^Shantou University Medical College, Shantou, China

## Abstract

**Purpose:**

To evaluate the clinical outcome of the three-random-point (TRP) marking method for toric intraocular lens (IOL) alignment using the iTrace aberrometer (Tracey Technologies Corp., Houston, TX). *Setting*. Department of Ophthalmology, Guangdong Eye Institute, Guangdong Academy of Medical Sciences, Guangdong Provincial People's Hospital, Guangzhou, China.

**Design:**

Prospective, randomized comparative trial.

**Method:**

Thirty eyes of 30 patients undergoing cataract surgery with coexisting corneal astigmatism of over 1.0 D were included in this study. All patients were prospectively randomized into the TRP marking group or slit-lamp horizontal meridian (SHM) marking group. TRP marking involved marking three points randomly in the corneal limbus of the patients and accurately marking the horizontal meridian was not required. The follow-up duration was 3 months after cataract surgery.

**Results:**

Fifteen eyes of 15 patients were in the TRP marking group and 15 eyes of 15 patients in the SHM marking group. There was no statistically significant difference in BCVA, UDVA, preexisting corneal astigmatism, or residual astigmatism between the groups before or after surgery (*P* > 0.05). The mean toric IOL misalignment was lesser but without significance in the TRP marking group than in the SHM marking group after 3 postoperative months (2.66° ± 1.42° versus 3.29° ± 1.67°; *P*=0.295).

**Conclusion:**

The TRP marking method using the iTrace aberrometer is simple and accurate for preoperative marking of toric IOLs. It can eliminate the potential systematic errors resulting from varying head positions during the preoperative keratometry measurement and from manual marking.

## 1. Introduction

It has been estimated that 30% of patients with cataract have preexisting astigmatism of over 0.75 D; 8% of the eyes have corneal astigmatism of over 2.00 D; and 2.6% of the eyes have corneal astigmatism of over 3.00 D [1]. Because persisting astigmatism can decrease the visual acuity and the vision quality of patients after cataract surgery, predictable correction of the preexisting corneal astigmatism is critical and popular. Several surgical techniques, such as limbal relaxing incisions, peripheral corneal relaxing incisions, and excimer laser surgery, are used to eliminate or decrease coexisting astigmatism in patients with cataract [2]. Toric intraocular lens (IOL) implantation during cataract surgery was more widely used owing to its reliability and effectiveness [3, 4].

Precise preoperative limbal marking is crucial for an accurate alignment of toric IOLs [5]. When the patient is changed from the standing or sitting position to the supine position, cyclotorsion of the eye can cause misalignment. The average cyclotorsion of the eye when the patient is changed from the upright position to the supine position is approximately 2°–4° but can be up to 15° [6, 7]. Manual methods for horizontal meridian limbal marking were commonly used preoperatively, including the slit-lamp marking, surgeon's direct visual marking, bubble marker-assisted method, pendular marker-assisted method, and tonometer marking. All manual methods for corneal marking can cause errors. Accurate preoperative marking of the eye with the patient in the sitting position can minimize the intraoperative misalignment errors. A digital image-guidance system used during toric IOL surgery is more reliable and precise than manual marking [8]. However, using a digital image-guidance system has disadvantages of high cost and need for special equipment. The aim of this study was to introduce the three-random-point (TRP) marking method performed using the iTrace system (Tracey Technologies Corp. Houston, TX).

## 2. Patients and Methods

All patients were randomly divided into the TRP marking group (15 eyes) and slit-lamp horizontal meridian (SHM) marking group (15 eyes). Randomization was performed using computerized random number tables. Written informed consent was obtained from the patients. The study complied with the principles laid down in the Declaration of Helsinki and was approved by the Ethics Committee of Guangdong Provincial People's Hospital. Uncorrected distance visual acuity (UDVA) and best-corrected visual acuity (BCVA) were recorded before and after surgery. The visual acuity was converted to the logarithm of the minimum angle of resolution. The axis length was measured with IOLMaster 500 (Carl Zeiss Meditec). Keratometry was performed using the iTrace aberrometer during corneal limbal marking. The patients with previous intraocular surgery, corneal opacities, irregular astigmatism, glaucoma, macular disease, or diabetic retinopathy were excluded.

The surgeries were performed by a single surgeon (HZ) as a routine procedure. An AcrySof® Toric IOL (Alcon Laboratories, Inc., Fort Worth, TX, USA) was implanted. The alignment axis of the toric IOL was obtained with the Calculation Tool software (version 3.2.4) provided on the Alcon website (http://www.acrysoftoriccalculator.com).

### 2.1. TRP Marking Technique

**Figure 1:**
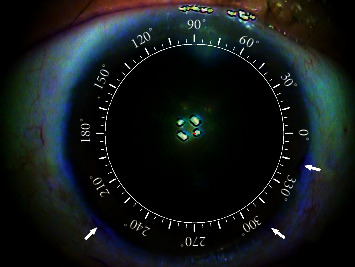
Corneal topography and anterior segment images are captured simultaneously with the iTrace system. Three random points are marked in the corneal limbus with a staining blunt needle. The arrows show the points marked randomly.

**Figure 2:**
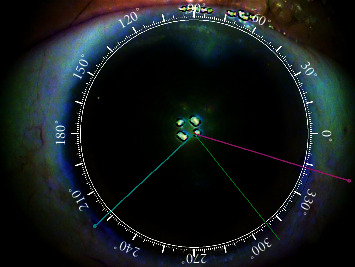
The Zaldivar Toric Caliper is used to measure the axis at which the marked points are located. The axis values corresponding to the three marked points are 223° (43°), 309° (129°), and 343° (163°), respectively (only two axis lines can be shown simultaneously. The third axis line needs to be measured separately).

**Figure 3:**
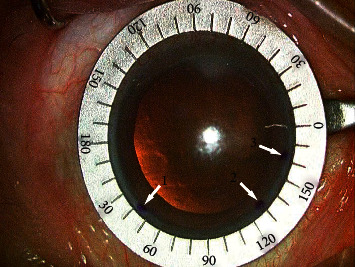
During cataract surgery, the Wallace Mendez Degree Gauge (Bausch and Lomb, Inc.) is placed aiming at the three marked points based on the axial value obtained with the Zaldivar Toric Caliper. Arrows 1, 2, and 3 are corresponding to the axis values of 223° (43°), 309° (129°), and 343° (163°), respectively.

After applying topical anesthesia, three points were marked randomly in the corneal limbus with a blunt staining needle and the patient in the upright position. Corneal topography and anterior segment images were captured with the iTrace system (Tracey Technologies Corp. TX, USA) (Figure 1). The Zaldivar Toric Caliper was used, and the center of the cornea was connected with the marked points in the limbus (Figure 2). The values of the marked meridian could be read using a caliper. During cataract surgery, the Wallace Mendez Degree Gauge (Bausch & Lomb, Inc.) was placed at the three marked points based on the Zaldivar Toric Caliper measurement (Figure 3).

### 2.2. SHM Marking Technique

In the SHM marking group, patients were seated in front of a slit lamp with the chin and forehead fixed. The slit of the slit-lamp (Haag-Streit AG) was turned in the horizontal position. Subsequently, the slit was focused on the center of the corneal apex, and the horizontal meridian was marked in the limbus by scratching the cornea with a blunt staining needle.

### 2.3. IOL Misalignment Evaluation

To examine the accuracy of the alignment of IOL, images of the anterior segment were captured after full mydriasis and analyzed using the iTrace aberrometer system after 3 postoperative months. Toric IOL misalignment was evaluated based on the difference between the target axis and the actual axis and measured using the Zaldivar Toric Caliper by a masked observer. The values of IOL alignment were measured using the Zaldivar Toric Caliper.

### 2.4. Statistical Analysis

Statistical analysis was performed with SPSS, version 19.0 (SPSS, Inc., Chicago, IL, USA). Values are expressed as mean ± standard deviation. The Mann–Whitney *U* test was used for comparison of between-group variables. *P* value <0.05 was considered statistically significant.

## 3. Results

This study included 30 eyes of 30 patients undergoing cataract surgery with coexisting corneal astigmatism of over 1.0 D. The TRP marking group included nine women and six men, with a mean age of 70.07 ± 7.38 years (range: 64 to 84 years). The SHM marking group included seven women and eight men, with a mean age of 72.8 ± 6.64 years (range: 61 to 85 years). No significant differences in preoperative corneal astigmatism were noted between the groups.  Table 1 shows the preoperative and postoperative outcomes in both the groups. There was no difference in UDVA or CDVA between the groups pre- or postoperatively. After 3 postoperative months, toric IOL misalignment was lesser but without significance in the TRP marking group than in the SHM marking group (2.66 ± 1.42 versus 3.29 ± 1.67; *P*=0.295).

## 4. Discussion

Errors in alignment can occur at several stages in cataract surgery and toric IOL implantation, including preoperative corneal biometry measurement, preoperative referent limbal marking, alignment on the intended meridian of toric IOL, and postoperative IOL rotation. For toric IOLs, every deviation of 3° from the intended meridian results in an astigmatic undercorrection of approximately 10% [9]. The preoperative corneal marking is an initial and crucial procedure for the accurate alignment of toric IOLs. Because the preoperative mark is used to identify the desired meridians for the incision and IOL alignment, it is considered to cause toric IOL deviation or misalignment more easily than other procedures [10]. Various methods have been proposed to obtain an accurate marking before the implantation of a toric IOL. The computer-assisted marker system for toric IOLs, facilitating the alignment of the patient's eye and tracking the image in real time, showed better results than manual marking [8]. Manual marking techniques assisted with a slit lamp or calibration device are used widely owing to the simplicity, reproducibility, and ease of use.

Preoperative limbal marking aims to mark the horizontal meridian (0°–180°) with the patient in the sitting position. Manual limbal horizontal marking has four sources of potential errors. The first source of error is the horizontal reference meridian error from the rotational deviation. Woo et al. performed a prospective comparative study to assess the accuracy of three horizontal meridian marks using the iTrace system and found that the average rotational deviation of horizontal marking ranged from −0.66° to 2.2° [10]. Farooqui et al. compared the accuracy of a bubble marker with that of a pendulum marker and found that the mean absolute alignment error for both techniques was similar and less than 3° [11]. Igarashi et al. assessed the horizontal meridian misalignment of limbal marking under a slit-lamp microscope and showed the axis misalignment by an average of 3.4° to 6.9° [12]. The second potential source of error could be the eye fixation and head position as the patient was sitting with the head positioned straight while being subjectively assessed by the examiner during the marking procedure. The third potential source of error is the difference in head positions with different devices at the time of preoperative keratometry measurement and marking the reference axis shortly before surgery. The fourth potential source of error is the vertical height error from the corneal center and mark line [10]. The alignment errors led to a reduction in the effectiveness of astigmatism correction, which was not necessarily negligible. Precisely making the reference is an essential step in achieving the desired effect of astigmatism correction.

The marking axis of the TRP marking method was measured with the Zaldivar Toric Caliper in the iTrace aberrometer system. The Zaldivar Toric Caliper in the iTrace system was used by Woo et al. to assess the accuracy of three different meridian marking methods. It has helped cataract surgeons confirm that the toric IOL is precisely positioned [10]. The TRP marking method using the iTrace aberrometer system has three advantages. First, three points are marked in the corneal limbus randomly, so marking the point precisely through the horizontal meridian is not necessary. Second, both corneal biometry measurement and corneal limbal marking were performed using the iTrace aberrometer, so it could eliminate the potential error resulting from varying head positions during preoperative keratometry measurement and marking the reference line under a slit lamp. Third, the corneal center overlapping with the marking point of TRP marking could help locate the Wallace Mendez Degree Gauge during surgery. Our result also suggested that the TRP marking method was comparable to the slit-lamp horizontal meridian marking method. A similar method not requiring the horizontal axis was introduced by Alex. In that method, a simple steep axis is marked using a corneal analyzer (OPD III scan). The technique requires a random reference mark in the limbus and avoids the potential error from varying positions of corneal measurement and limbal marking [13].

The main limitation of TRP marking is the machine dependence. However, this method is simple, quick, and accurate. The horizontal meridian does not need to be marked under a slit lamp. This eliminates the potential error from varying head position and cyclotorsion. The other limitation is the relatively small sample size. We will expand the sample size to further evaluate the new method.

### 4.1. What Was Known

Accurate axis marking is essential for astigmatic correction during cataract surgery with toric IOL. Manual methods for horizontal meridian limbal marking were commonly used preoperatively, but there are unavoidable errors. Improving the marking method can reduce the errors.

### 4.2. What This Paper Adds

The TRP method can mark the points randomly in a sitting or supine position. The three-point markers can avoid error from varying positions of corneal measurement and limbal marking. The three-random-point marking method is a simple, quick, and accurate method for toric IOL implantation.

## Figures and Tables

**Table 1:** Comparison of pre- and postoperative outcomes between the three-random-point marking group and the slit-lamp horizontal meridian marking group.

Parameter	TRP marking group	SHM marking group	*P* value^*∗*^
Preexisting corneal astigmatism cyl (D)	2.31 ± 0.61	2.24 ± 0.42	0.727
Postop cyl (D)	0.41 ± 0.17	0.47 ± 0.13	0.240
Preop UDVA (logMAR)	0.52 ± 0.17	0.62 ± 0.22	0.169
Preop CDVA (logMAR)	0.35 ± 0.10	0.41 ± 0.12	0.172
Postop UDVA (logMAR)	0.13 ± 0.07	0.10 ± 0.05	0.125
Postop CDVA (logMAR)	0.11 ± 0.07	0.07 ± 0.05	0.06
Toric IOL misalignment (°)	2.66 ± 1.42	3.29 ± 1.67	0.295

TRP = three-random-points; SHM = slit-lamp horizontal meridian; cyl = cylinder; UDVA = uncorrected distance visual acuity; CDVA = corrected distance visual acuity; logMAR = logarithm of the minimum angle of resolution; IOL = intraocular lens. ^*∗*^Statistically significant differences, *P* < 0.05.

## Data Availability

All the data generated or analyzed during this study are available from the corresponding author on reasonable request.
